# Numerical Simulation of Passage of a Neutrophil through a Rectangular Channel with a Moderate Constriction

**DOI:** 10.1371/journal.pone.0059416

**Published:** 2013-03-20

**Authors:** Atsushi Shirai, Sunao Masuda

**Affiliations:** 1 Institute of Fluid Science, Tohoku University, Sendai, Miyagi, Japan; 2 Graduate School of Engineering, Tohoku University, Sendai, Miyagi, Japan; University of California, Irvine, United States of America

## Abstract

The authors have previously presented a mathematical model to predict transit time of a neutrophil through an alveolar capillary segment which was modeled as an axisymmetric arc-shaped constriction settled in a cylindrical straight pipe to investigate the influence of entrance curvature of a capillary on passage of the cell. The axially asymmetric cross section of a capillary also influences the transit time because it requires three-dimensional deformation of a cell when it passes through the capillary and could lead to plasma leakage between the cell surface and the capillary wall. In this study, a rectangular channel was introduced, the side walls of which were moderately constricted, as a representative of axially asymmetric capillaries. Dependence of transit time of a neutrophil passing through the constriction on the constriction geometry, i.e., channel height, throat width and curvature radius of the constriction, was numerically investigated, the transit time being compared with that through the axisymmetric model. It was found that the transit time is dominated by the throat hydraulic diameter and curvature radius of the constriction and that the throat aspect ratio little affects the transit time with a certain limitation, indicating that if an appropriate curvature radius is chosen, such a rectangular channel model can be substituted for an axisymmetric capillary model having the same throat hydraulic diameter in terms of the transit time by choosing an appropriate curvature radius. Thus, microchannels fabricated by the photolithography technique, whose cross section is generally rectangular, are expected to be applicable to *in vitro* model experiments of neutrophil retention and passage in the alveolar capillaries.

## Introduction

The function of the lung is to oxygenate the blood and to remove carbon dioxide. For this purpose, the lung is made up of an aggregation of alveoli and a dense network of capillaries surrounding individual alveoli. It is known that approximately one half of the neutrophils, the most common type of leukocyte, stop at least once during their journey through the lung [1], [2] and that it takes some neutrophils more than 20 min. to pass through the pulmonary capillary bed [3], while erythrocytes flow through the lung in a few seconds with little or no observable delay [4], [5]. This retention leads to a 40- to 100-times higher concentration of neutrophils in the pulmonary capillary bed than in systemic large vessels [6], [7]. These highly concentrated neutrophils are thought to help the lung to effectively eliminate foreign infectious substances brought in with inhaled air. Such sites where neutrophils exist at a high concentration are often referred to as marginated pools. Marginated pools are also seen in post-capillary venules of most organs, but the mechanism of the retention is largely different. That is, in the pulmonary capillaries, whose diameter is smaller than that of neutrophils, they are retained in the capillaries due to their low deformability as compared with that of erythrocytes [8], while margination occurs as rolling of neutrophils on the vessel wall mediated by bindings of selectins and their ligands in the post-capillary venules [9]–[11]. Therefore, in the pulmonary capillaries, deformation characteristics of neutrophils, in addition to the highly interconnected structure of the capillary network, are thought to account for their longer transit time and the resultant high concentration.

It is essential to clarify transit characteristics of neutrophils as they pass through the pulmonary capillary bed for the fundamental understanding of their behavior and functions in their immune response in the lungs. For this purpose, several models have been proposed on the passage of a neutrophil through individual capillaries and the structure of the capillary bed. As a mathematical model of passage of a neutrophil through a capillary segment, Huang et al. [12] presented a model to predict the transit time based in part on results of micropipette aspiration experiments [13] and in part on theoretical prediction of the entrance of a cell into a micropipette [14]. Though this model took into account the effects of the driving force of the cell and the minimum radius of the capillary on the transit time, it was based on studies using blunt-ended micropipettes. Bathe et al. [15] noted that the capillary geometry could significantly influence neutrophil transit time and that the effects of entrance curvature and lack of axisymmetry were important to consider. Bathe et al. [15] and Shirai et al. [16], [17] subsequently presented more general expressions for predicting neutrophil transit time, assuming a capillary as an axisymmetric moderate constriction in a straight pipe to take into account the effect of the entrance curvature of the capillary. The influence of the lack of axisymmetry of the capillary, however, has not been investigated. The lack of axisymmetry requires three-dimensional deformation of a cell in passing through a capillary and could lead to plasma leakage through clearances between the cell surface and the capillary wall due to a lack of a good seal between them, thus affecting transit time.

For the alveolar capillary network, there are two representative morphometric models. The first one is the sheet-flow model in which the capillary network consists of two membranes held apart by a number of more or less equally spaced posts [18]–[20]. The second one is the tube-flow model in which the capillary network is considered to be a hexagonal network of short cylindrical tubes [21], as evidenced by precise SEM observations of alveolar capillaries of young Sprague-Dawley rats showing that the pulmonary capillaries are tubular and not different from other capillary beds except in density [22]. Thus, capillary network models to investigate the passage of neutrophils have fundamentally been based on the tube-flow model [12], [23], [24], although in reality, the capillary segments are random in their cross-sectional shape.

Recently, cell-scale microchannels fabricated by the photolithography technique have been employed for observation of cell deformation and travel characteristics [25], as well as for trapping some specific cells from blood for examination or for developing a μ-TAS (Micro-Total Analysis System). The photolithography technique is expected to be applicable to the fabrication of the complicated two-dimensional interconnected structure of the capillary network and geometry of individual capillaries to mimic the alveolar capillary network, which would facilitate *in vitro* observations of blood perfusion or neutrophil retention in the capillaries. The cross-sectional shape of those channels is, however, rectangular like that of the vessel lumen of the sheet-flow model through which blood flows, and thus it is necessary to elucidate the transit characteristics of neutrophils through such rectangular channels for application of the microchannels to experiments.

Therefore, in this study, a capillary segment was modeled as a rectangular channel with a moderate constriction as a representative of axially asymmetric capillaries, this segment being extracted from the vessel lumen of the sheet-flow model. The influence of channel geometry, i.e., channel height, throat width and curvature radius of the constriction, on passage of a neutrophil through the constriction was numerically investigated, comparing transit time of the cell through the constriction with that of the former axisymmetric model.

## Methods

In this section, previous and present models of a neutrophil transit through a narrow channel are briefly introduced. See the cited references for details of the modeling and numerical procedure [15], [16].

### Neutrophil Model

Various rheological models have been proposed for a neutrophil: a homogeneous sphere composed of standard viscoelastic material [26], [27], a Newtonian liquid drop with a shell-like surface layer under tension [14], [28], a model in which the inner Newtonian liquid is replaced by a Maxwell fluid [29], [30] or by a power-law fluid [31], and a three-layer or compound drop model in which the cortical membrane of the lipid bilayer, the cytoplasm and the nucleus are taken into consideration [32]–[34]. Of these models, the authors chose the Maxwell fluid model encapsulated by a thin elastic membrane with a constant cortical tension because this model captures both elastic solid-like short time-scale behavior of the cell and its viscous fluid-like long time-scale behavior in aspiration into or expulsion from a micropipette, which would be a dominant feature of the cell that characterizes the transit time of a cell through a capillary segment.

Stress and strain in the homogeneous cell interior are related through the linear Maxwell constitutive law written as
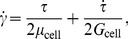
(1)where µ_cell_ is the constant coefficient of viscosity, *G*_cell_ is the constant elastic shear modulus and a superimposed dot denotes time differentiation of the variable. To fit the force-displacement relations in the “cell poking” experiment of neutrophils [35], 31 Pa·s of µ_cell_ and 186 Pa of *G*_cell_ were adopted [15], assuming a constant cortical tension of 31 pN/µm [29], [35]. Poisson’s ratio of the interior was determined to be 0.4999 as in former studies [15]–[17] and the cell was determined to be a sphere with a radius of *R*_cell_ = 4 µm at a steady state [36]. For the lipid bilayer of the cortical membrane, its Young’s modulus and thickness were determined to be 10^−4^ Pa and 1 nm, respectively, its bending stiffness being neglected because the bending stiffness of the membrane strongly affects the deformation of the cell only in the case that the cell is aspired into an extremely narrow pipette whose inner diameter is less than 1 µm [37].

### Axisymmetric Capillary Model

**Figure 1:**
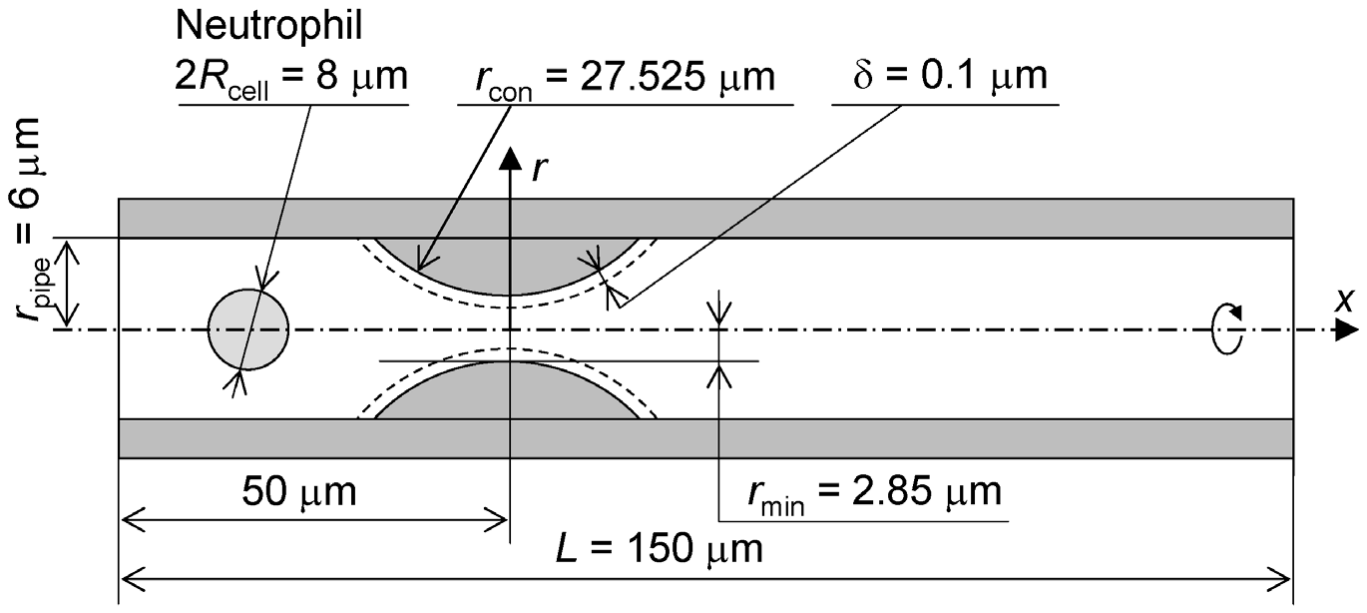
Schematic of axisymmetric capillary model.

Figure 1 shows a schematic of the axisymmetric capillary model [15], [16] to compare transit time of the neutrophil through that model with such transit time through the rectangular channel model mentioned in the next section. This model is a straight cylindrical pipe *L* = 150 µm in length and *r*_pipe_ = 6 µm in radius. An axisymmetric arc-shaped constriction of *r*_con_ = 27.525 µm in the curvature radius and *r*_min_ = 2.85 µm in the throat radius is employed to position its throat 50 µm downstream from inlet of the pipe. In this figure, the constricted region correlates with a capillary segment. Here, an imaginary wall exists δ = 0.1 µm apart from the constriction surface, as illustrated by broken lines in the figure, to prevent collapse of the finite elements of the fluid region in the numerical simulation, and thus, the throat radius of the imaginary wall is 2.75 µm and the length of the constricted region is 26 µm. The neutrophil deforms along the imaginary wall with no friction, while plasma flow penetrates the imaginary wall. This geometry of the capillary was designed taking into consideration the cylindrical posts in the sheet-flow model [18]–[20], a microscopic photograph of an alveolar capillary network [38], and the average diameter of 5.5 µm [22], [39]. Friction of the cell on the imaginary wall was neglected because the dominant cause of the delay in passage of a cell through a capillary is thought to be the low deformability of the cell.

### Rectangular Channel Model

**Figure 2:**
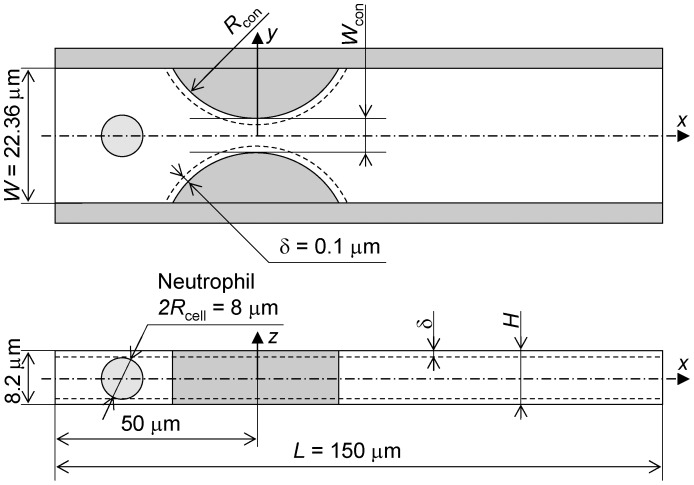
Schematic of rectangular channel model.

Figure 2 shows a schematic of the rectangular channel model. This model was designed to simulate a segment of the vessel lumen of the sheet-flow model [18]–[20] through which blood flows. Therefore, the constricted region correlates with a capillary segment. The channel is *W* = 22.36 µm in width, *L* = 150 µm in length and *H* in channel height. The *x*-axis is at the center of the channel. Each of the side walls is an arc-shaped constriction whose curvature radius is *R*_con_, which mimics the posts in the sheet-flow model, making the throat width *W*_con_ and positioning the throat 50 µm downstream from inlet of the channel. Here, as in the axisymmetric capillary model, imaginary walls are set δ = 0.1 µm apart from the side walls of the constriction, ceiling and floor of the channel as illustrated by broken lines in the figure. *H*, *R*_con_ and *W*_con_ were changed to examine their contributions to the transit time of a cell through the constriction. For the condition of *H* <8.2 µm, the channel height at the inlet was fixed at 8.2 µm and gradually decreased to *H* in the region −40.17 µm ≤ *x* ≤ −35 µm to compress the cell before it reached the constriction.

### Numerical Procedure

Plasma which filled the channel was assumed to be an incompressible Newtonian fluid with viscosity of 1.2×10^−3^ Pa·s and density of 1.03×10^3^ kg/m^3^. A cell which was initially spherical was buoyant in the plasma on the *x*-axis and it moved with the plasma driven by a pressure drop Δ*P* of 40 Pa across the channel. A non-slip boundary condition of the plasma was applied to the channel walls and the cell surface.

Computation was performed using commercial finite element solver ADINA version 8.2 (Watertown, MA). For the axisymmetric capillary model, the cell was discretized in space using four-node quadrilateral axisymmetric elements. The plasma region was discretized in space using three-node triangular axisymmetric elements. For the rectangular channel model, both the cell and the plasma regions were discretized using four-node tetrahedral elements. The updated Lagrangian Hencky kinematic formulation was used to analyze the cellular response and the constant function method was used to satisfy the nonlinear contact conditions. The finite element governing equations were solved incrementally in time using the Euler backward method for the fluid-structure interaction. For the plasma flow, the elements interpolated pressure bi-linearly and employed an additional nonlinear bubble function to interpolate velocity.

The fluid-structure interaction (FSI) analysis capability of ADINA was used to examine the moving boundary between the cell and the plasma. The solution obtained was fully coupled, in the sense that continuity of the kinematic conditions of displacement, velocity and acceleration across the interface, as well as the continuity of the shear stress and discontinuity of normal stress due to the cortical tension, was satisfied during the entire analysis. The time inclination step was determined to be 0.001 s, which was a sufficiently fine time step in the former studies [16], [17].

## Results

### Passage through the Rectangular Channel Model

**Figure 3:**
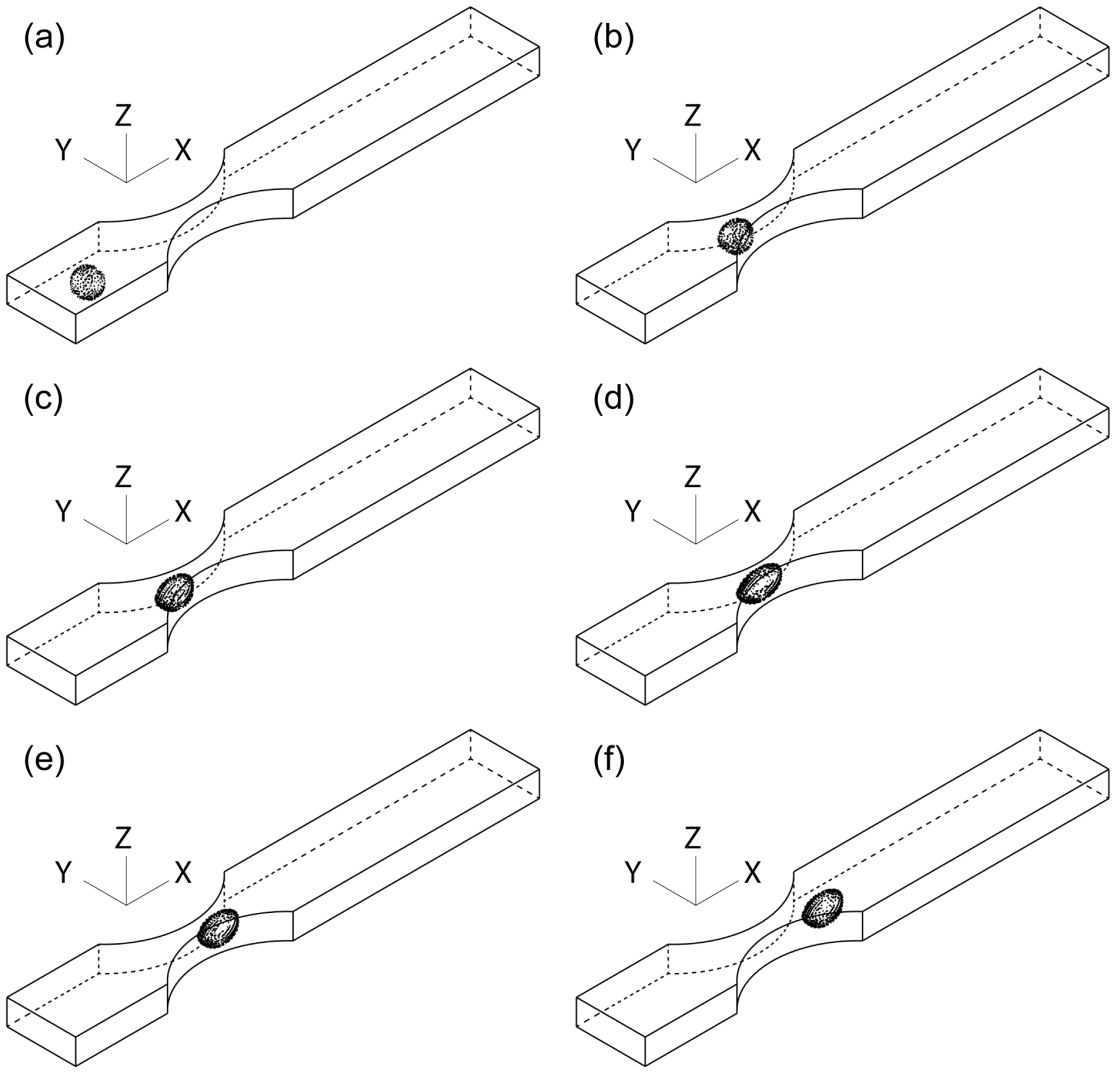
Passage of a neutrophil through a rectangular channel. *H* = 8.20 µm, *R*_con_ = 27.525 µm and *W*_con_ = 4.368 µm at Δ*P* = 40 Pa. (a) *t* = 0.0 s, (b) *t* = 0.1 s, (c) *t* = 0.5 s, (d) *t* = 0.84 s, (e) *t* = 0.855 s and (f) *t* = 0.87 s.

Figure 3 shows an example of the successive images of passage of a neutrophil through the constriction of a rectangular channel model of *H* = 8.20 µm, *R*_con_ = 27.525 µm and *W*_con_ = 4.368 µm at Δ*P* = 40 Pa. This condition was adopted as the control throughout this study. It should be noted that height and width of the channel lumen through which a cell traveled was 0.2 µm smaller than these values due to the imaginary walls set δ = 0.1 µm inside of the lumen. During the passage, the cell was first deformed to some extent by its elastic component followed by a subsequent slow deformation by the viscous component. The cell was still deformed even after it was released from the throat of the constriction. It was confirmed, while the cell was being squeezed, that there were clearances between the cell surface and corners of the rectangular channel (termed gutters, hereafter). Plasma leakage through the gutters is thought to decrease the driving force of the cell by the pressure drop across the cell.

**Figure 4:**
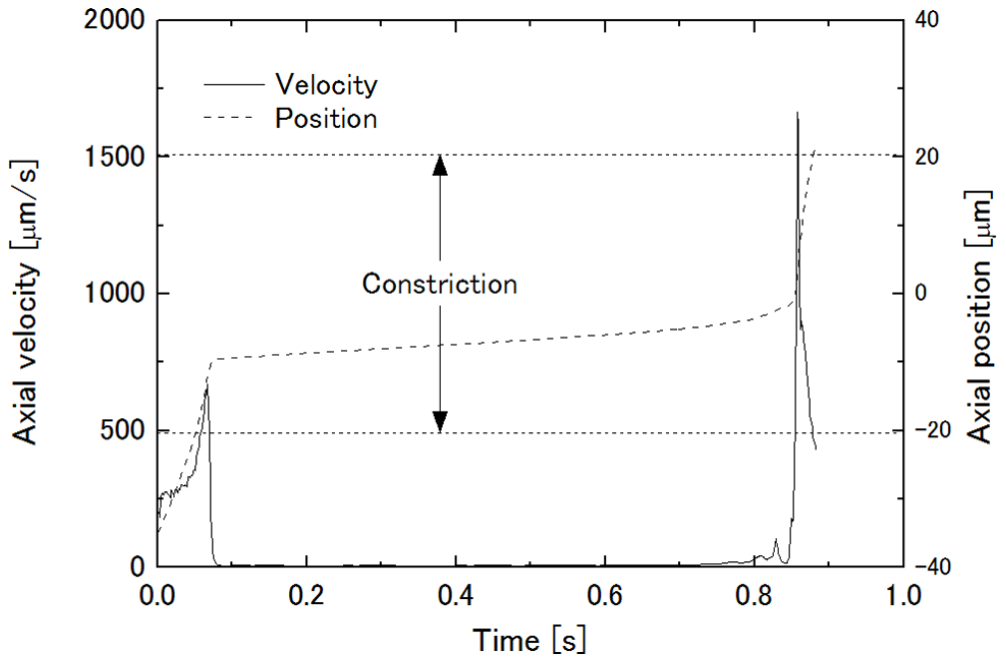
Time variation of axial velocity and position of cell shown in Figure 3.

Figure 4 shows the time variation of axial velocity and the position of the midpoint of the leading and trailing ends of the cell shown in Figure 3. It is obvious that there are velocity peaks just before the cell came in contact with the side walls of the constriction and when the cell passed through the throat of the constriction, and that most of the passage period was spent in the upstream half of the constriction where the cell was being squeezed. Thus, it can be said that transit time of the cell through the constriction is characterized by its deformation characteristics because the cell slips on the imaginary wall with no friction.

Transit time of a cell through a constriction of the axisymmetric capillary model in the former studies [15]–[17] was defined as the period between the time when the leading end of the cell reached the upstream end of the constricted region and the time when the trailing end passed the downstream end of the constricted region. In this study, however, fluid elements were distorted and collapsed, resulting in a breakdown of the computation before the cell passed the downstream end of the constricted region of the rectangular channel model under certain conditions. Thus, in this study, as a compromise, the period between the two velocity peaks was chosen to be the transit time, assuming the discrepancy between the transit times between the former and the present definitions to be small. This is because cell velocity in the straight region was >250 µm/s (see the axial velocity around 0.0 s in Fig. 4) and the cell velocity in the downstream half of the constriction after the cell was expulsed from the throat of the constriction should exceed this value due to channel width being narrower than that of the straight region.

### Validation of Computation

In the previous section, the number of elements was 2,935 for the neutrophil and 20,521 for the plasma region, the available upper limit of the computer. To examine the dependence of the number of elements on the transit time, a number was chosen from 621, 1,198, 1,729 and 2,935 for the cell and from 9,839, 12,400, 16,014 and 20,251 for the plasma region. When the number of elements of the plasma region was changed with that of the cell being fixed at 2,935, the transit time was 0.105 s, 0.106 s, 0.106 s and 0.104 s, respectively. In contrast, when the number of elements of the cell was changed with that of the plasma region being fixed at 20,251, it was 0.140 s, 0.130 s, 0.102 s, and 0.104 s, respectively. Therefore, the number of elements of the cell should be 1,729 or more while that of the plasma region little affected the transit time. However, the finest mesh was chosen for both the cell and the plasma regions because control of the mesh sliding feature in the numerical simulation to prevent collapse of the elements was difficult with coarse mesh. Here, note that the number of elements of the plasma region changed about ±1% with the constriction geometry in the following simulations.

### Influence of Channel Height on Transit Time

**Figure 5:**
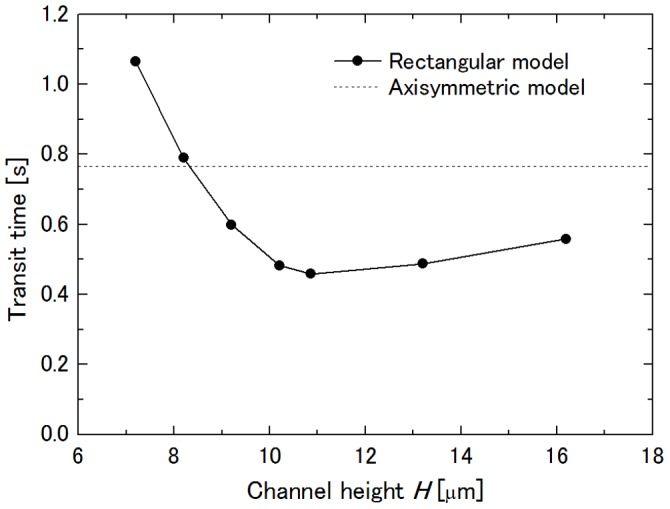
Influence of channel height *H* on transit time. *R*_con_ = 27.525 µm and *W*_con_ = 4.368 µm at Δ*P* = 40 Pa.

First, the channel height *H* was changed between 7.2 µm and 16.2 µm. Figure 5 shows the dependence of the transit time of a neutrophil on *H*. In this figure, the transit time decreases with the increase in *H*, the minimum transit time being at *H* = 10.86 µm, and then increases. From precise observation of the cell deformation during its passage, it was found that *H* = 10.86 µm was the height of the maximally deformed cell. Therefore, it can be said that the magnitude of cell squeezing decreases with the increase in *H* so that the cell can easily pass through the throat of the constriction in the range *H* ≤10.86 µm. In contrast, the pressure drop across the cell to drive it decreased due to the increased plasma leakage through the clearances between the cell surface and ceiling or floor of the channel when *H* increased more because the deformed cell shape was the same due to the absence of compression from the ceiling and the floor in this range. Here, for *H* to yield the minimum transit time, it must be decreased with the increase in *W*_con_ with which the height of the maximally deformed cell decreases.

### Influence of Throat Width on Transit Time

**Figure 6:**
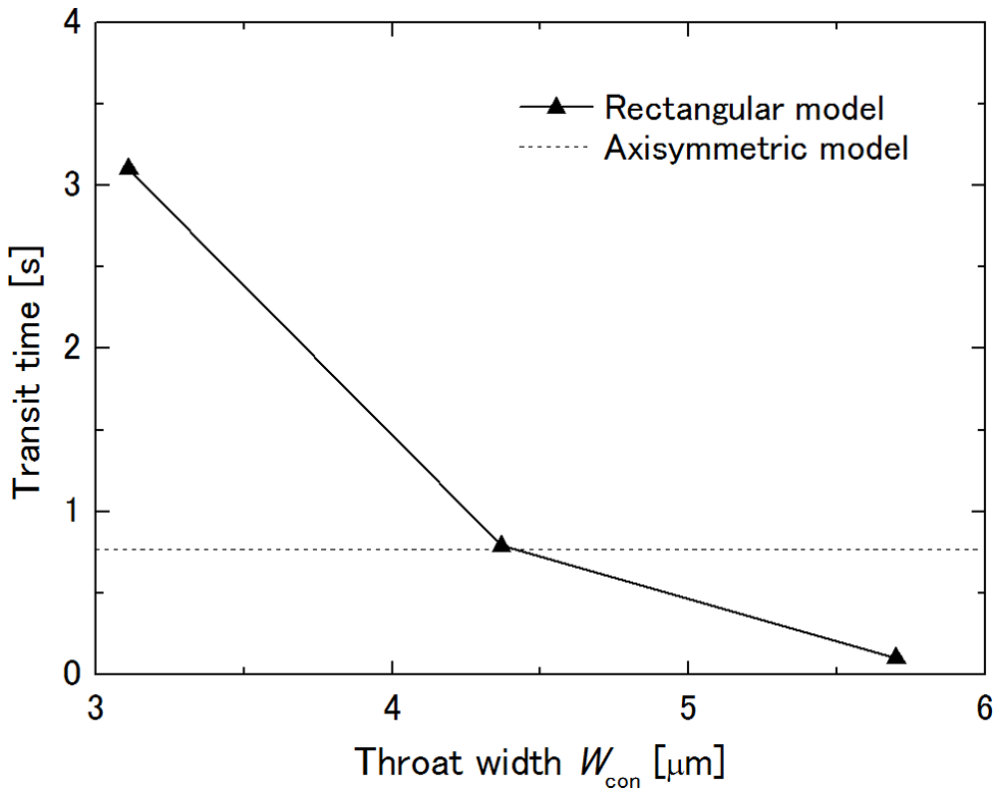
Influence of throat width of constriction *W*_con_ on transit time. *H* = 8.2 µm and *R*_con_ = 27.525 µm at Δ*P* = 40 Pa.

Dhadwal et al. [40] assumed the cross section of the vessel lumen between each pair of posts in the sheet-flow model to be elliptical and characterized the lumen by its hydraulic diameter. To enable examination of the influence of the throat width on the transit time of a cell through the constriction, the throat width, *W*_con_, of the constriction in the rectangular channel model was chosen to be 3.112 µm, which gives the same cross-sectional area, 4.368 µm, which gives the same hydraulic diameter, and 5.7 µm, which is the same width as the throat of the axisymmetric capillary model. Here, the height of the channel and curvature radius of the constriction were fixed at *H* = 8.2 µm and *R*_con_ = 27.525 µm, respectively. Figure 6 shows the dependence of the transit time on *W*_con_ together with the transit time through the axisymmetric capillary model. Since the simulation at *W*_con_ = 3.112 µm broke down when the cell had just been expulsed from the throat of the constriction before showing the second velocity peak, the transit time of the cell at this condition was estimated to be between 3.1 s and 3.2 s, taking into consideration the width of the velocity peaks of other conditions. The transit times of other conditions were 0.79 s for *W*_con_ = 4.368 µm and 0.10 s for *W*_con_ = 5.7 µm, while that in the axisymmetric capillary model was 0.77 s. This result implies that the rectangular channel model yields a transit time similar to that of the axisymmetric capillary model when the same throat hydraulic diameter is chosen.

### Influence of Throat Aspect Ratio on Transit Time

**Figure 7:**
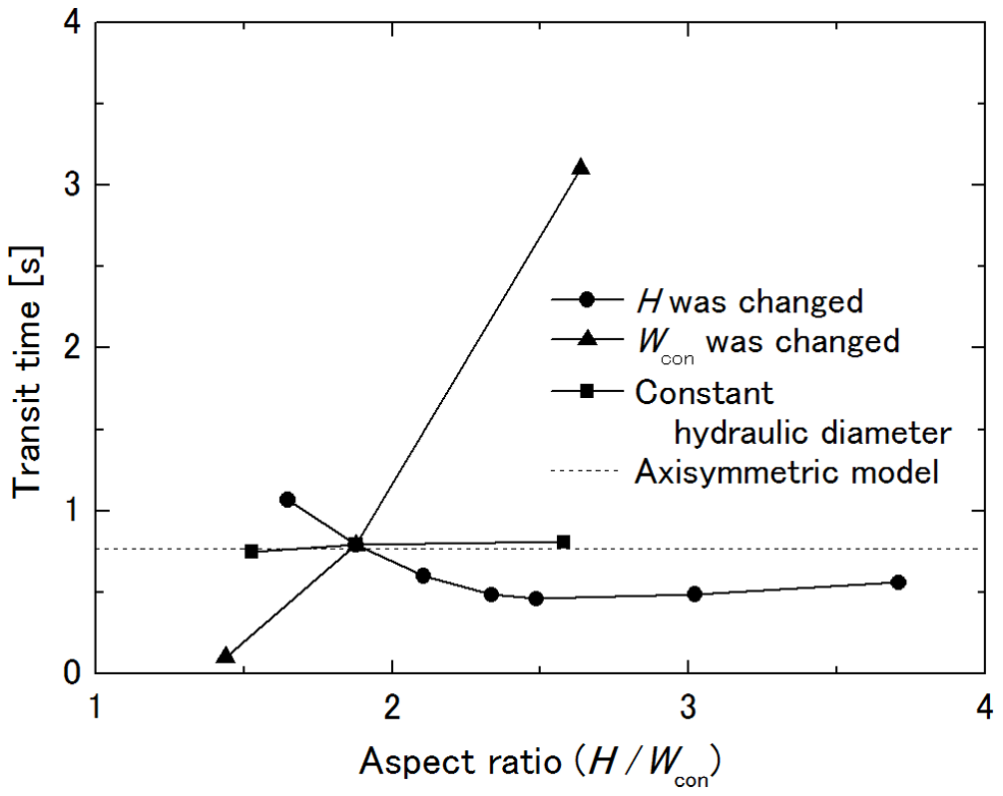
Influence of aspect ratio *H*/*W*_con_ on transit time. *R*_con_ = 27.525 µm at Δ*P* = 40 Pa.

The aspect ratio of the throat of the constriction *H*/*W*_con_ was changed in the manner listed in Table 1, keeping the same hydraulic diameter as that of the axisymmetric capillary model. Figure 7 shows the dependence of the transit time on the aspect ratio. For comparison, the results shown in Figures 5 and 6 are also plotted with respect to the throat aspect ratio. In this figure, it is obvious that the aspect ratio little influences the transit time as long as the throat hydraulic diameter is the same. The transit time was 0.75 s for No. 1, 0.79 s for No. 2 and 0.81 s for No. 3, and the discrepancy of the transit time from that of the axisymmetric capillary model of 0.77 s was within 5%. In this range of the aspect ratio, the cell in the constriction remained in contact with the ceiling and the floor as well as with the side walls of the constriction. Although not confirmed because the range of the introduced aspect ratios was the limit of stable computation, the transit time of a cell through an extremely thin or narrow constriction would be increased due to the decrease in the pressure drop across the cell to drive it, which was induced by increased plasma leakage through the clearances between the cell surface and the channel walls, as shown in Figure 5.

**Table 1:** Combination of channel height *H* and throat width of constriction *W*_con_.

No.	*H* [µm]	*W*_con_ [µm]	Aspect ratio
1	7.2	4.717	1.53
2	8.2	4.368	1.88
3	10.2	3.955	2.58

They have the same throat hydraulic diameter as that of the axisymmetric capillary model.

### Influence of Curvature Radius of Constriction on Transit Time

**Figure 8:**
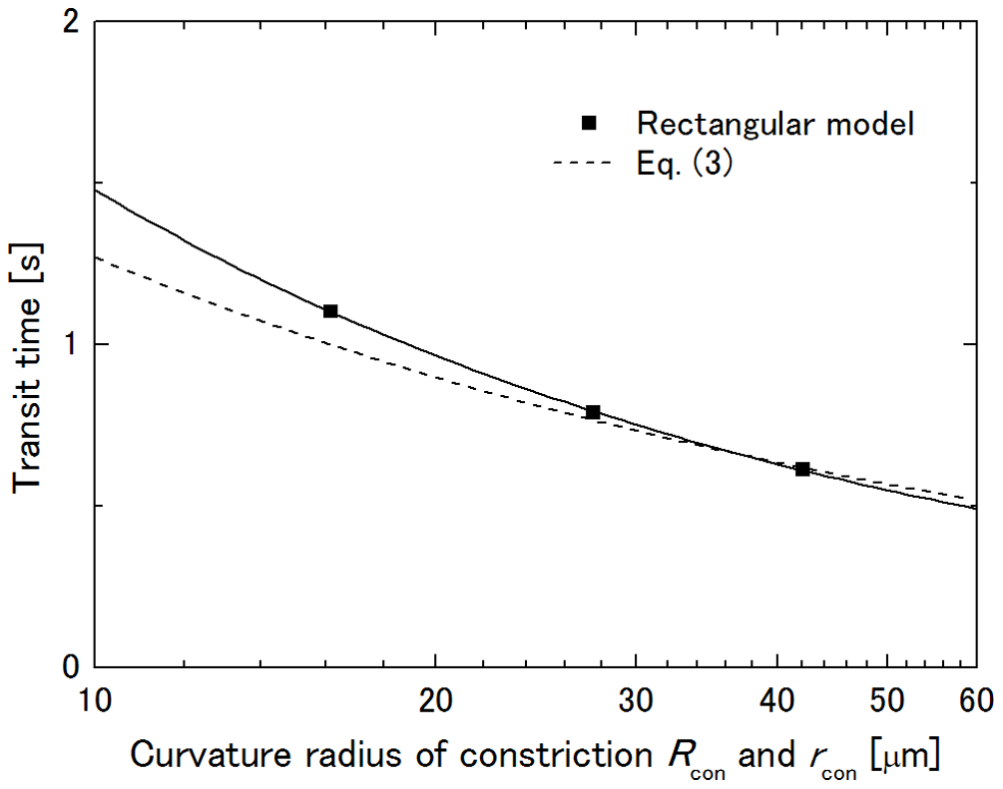
Influence of curvature radius of constriction *R*_con_ on transit time. *H* = 8.2 µm and *W*_con_ = 4.368 µm at Δ*P* = 40 Pa.

It has been shown in previous studies that, in addition to the throat radius of the constriction, the curvature radius of the constriction also influences the transit time, the transit time of a cell decreasing with the increase in the curvature radius of the constriction even though the total length of the constricted region increases [15], [16]. Thus, the curvature radius *R*_con_ of the constriction was chosen from 16.15, 27.525 and 42.15 µm following those studies. Figure 8 shows the dependence of the transit time on *R*_con_. For the rectangular channel model, the transit time *T* decreases with the increase in *R*_con_ and follows
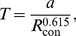
(2)while it follows
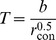
(3)for the axisymmetric capillary model [16]. Here, constants a and b are a = 6.10 s·µm0.615 and b = 4.02 s·µm0.5 in this condition, and the lines cross at 37.6 µm. This result implies that the deformation mechanism of the cell in its passage through the rectangular channel model is the same as that through the axisymmetric capillary model. The difference in the exponents of Rcon and rcon is considered to be due to the flat ceiling and floor of the channel and to plasma leakage through the gutters.

## Discussion

### Influence of Constriction Geometry on Transit Time

The present results indicate dependence of transit time of a neutrophil through a moderate constriction positioned in a rectangular channel with channel height *H*, throat width *W*_con_, and curvature radius *R*_con_ of the constriction.

The dependence of the transit time on *H* showed a local minimum at *H* = 10.86 µm, which was the height of the maximally deformed cell during passage through the constriction. As mentioned in the results section, a decrease in the transit time with an increase in *H* in the range *H* ≤10.86 µm is due to decreased magnitude of the cell squeezing. In the range *H* >10.86 µm, each pair of ceiling-side and floor-side gutters were united with each other, thus allowing greater plasma leakage, resulting in a decrease in the pressure drop across the cell to drive it. Comparing gradients of the transit time vs. *H* between these two ranges, the gradient in the former range is steeper than that in the latter, and thus, it can be said that the contribution of the change in the pressure drop is smaller than that in the viscous resistance to the cell deformation.

**Figure 9:**
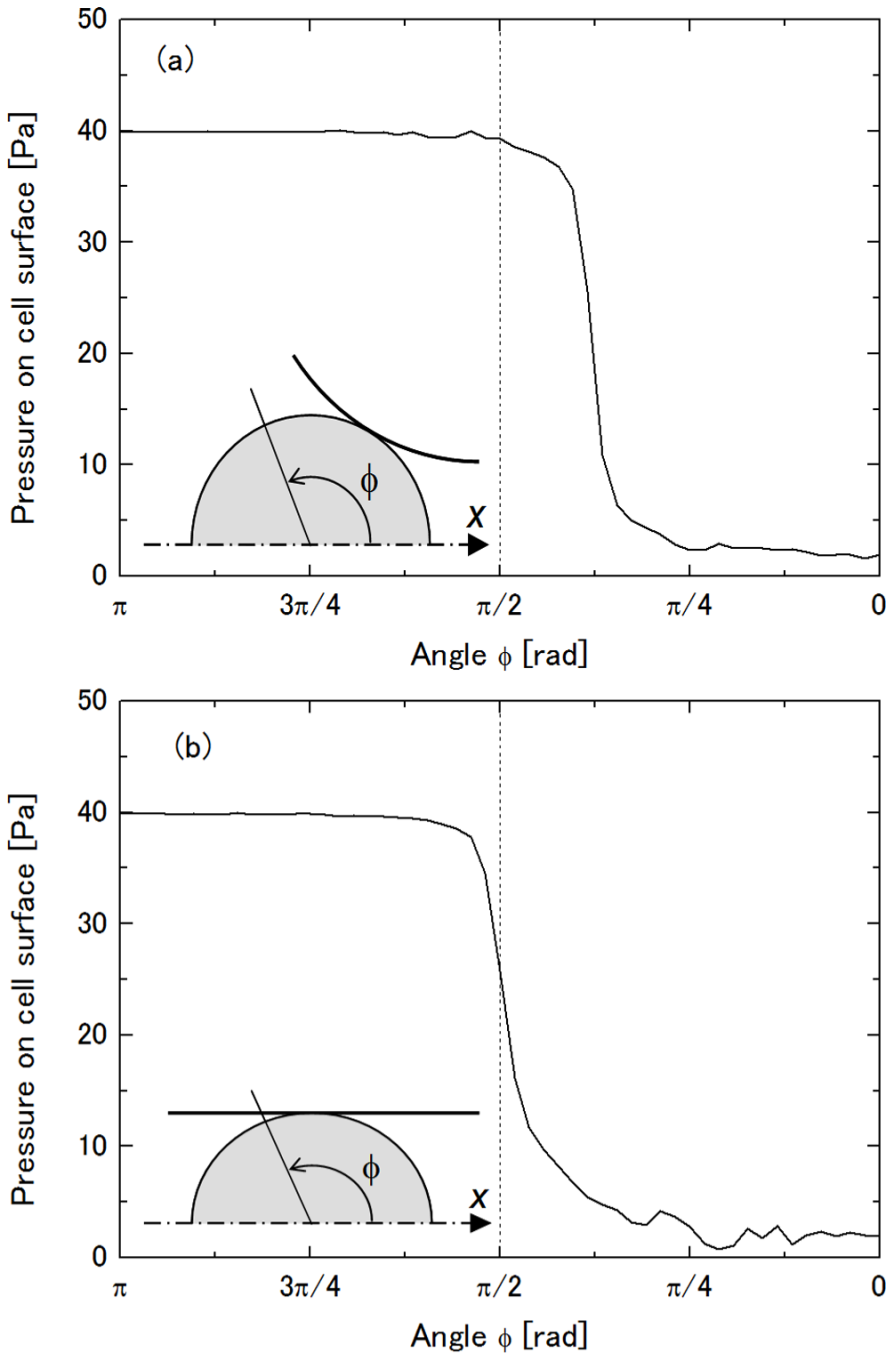
Pressure distribution on a rigid sphere stacked in a constriction of a rectangular channel [**42**]. (a) *x*-*y* plane and (b) *z*-*x* plane.

When *H* was constant, the transit time decreased with the increase in *W*_con_. Regarding the cause of the decrease in the transit time, three reasons can be considered in addition to the decreased deformation of the cell as in the case when *H* was changed. The first one is decreased length of the constricted region for the cell to be squeezed while it is passing through the constriction. That is, under a constant *R*_con_, the length of the constricted region along which the cell passes while being squeezed by the side walls of the constriction decreases with the increase in *W*_con_. For example, the axial distance from the throat of the constriction to the center of a spherical cell when it first comes in contact with the side walls of the constriction (length of cell squeezing, hereafter) is 12.4 µm at *W*_con_ = 3.112 µm, 10.8 µm at *W*_con_ = 4.368 µm and 8.80 µm at *W*_con_ = 5.7 µm. The second reason is change in the contact angle of the cell with the side walls. It is explained qualitatively as follows using the axisymmetric capillary model [16]. When a cell encounters the constriction, it first deforms to some extent due to its elastic component. Reaction stress τ applied from the constriction surface to the elastically deformed cell in the normal direction to the contact area is described as
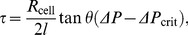
(4)where *l* is the width of the ring-shaped area of the elastically deformed cell in contact with the constriction surface, θ is the contact angle which is the angle of elevation of the cell from the *x*-axis to the center of the width of the contact area, Δ*P* is the pressure drop across the cell which plugs the constriction, and Δ*P*_crit_ is the critical pressure drop for a cell to be aspired into a micropipette [28]. Since θ increases and Δ*P*_crit_ decreases with the increase in throat radius of the constriction *r*_min_, the radial component of τ which squeezes the cell also increases. This result matches the finding by Trans-Son-Tay et al. [41], who showed that the entry rate of a droplet into a tapered micropipette decreases with increasing taper angle of the pipette. In the rectangular channel model, the stress which squeezes the cell applied from the side walls of the constriction depends on θ as well, and θ increases with an increase in *W*_con_, though the stresses from the ceiling and the floor are independent of θ. The third reason is the three-dimensional pressure distribution on the cell surface while it is in the constriction specific to the rectangular channel. Figure 9 shows pressure distribution on a rigid sphere with a diameter of 10 µm stuck in the constriction of a rectangular channel model [42]. In the cited study, the channel height was the same as the diameter of the sphere, and thus, the pressure distribution was qualitatively the same as those in Figure 6. In Figure 9, the angle φ where pressure drastically changes corresponds to the contact angle θ. Therefore, it changes with θ in the *x*-*y* plane due to the change of *W*_con_ while it is π/2, independent of *W*_con_ in the *z*-*x* plane. Figure 9 (a) indicates that an increase in θ increases the driving force of the cell by the pressure drop because the high pressure region in the range of φ<π/2 acts as a resistance to the passage of the cell. The decreased transit time with the increase in *W*_con_ is considered to be the result of a combination of those reasons. However, the transit time decreased with an increase in *R*_con_ though the length of cell squeezing increased: 8.60 µm at *R*_con_ = 16.15 µm, 10.8 µm at *R*_con_ = 27.525 µm and 13.2 µm at *R*_con_ = 42.15 µm, as shown in Figure 8. Since θ increases with the increase in *R*_con_ under the same *W*_con_, it can be said that the magnitude of cell squeezing and the contact angle rather than the length of cell squeezing dominate the transit time as in the former studies [15], [16].

**Figure 10:**
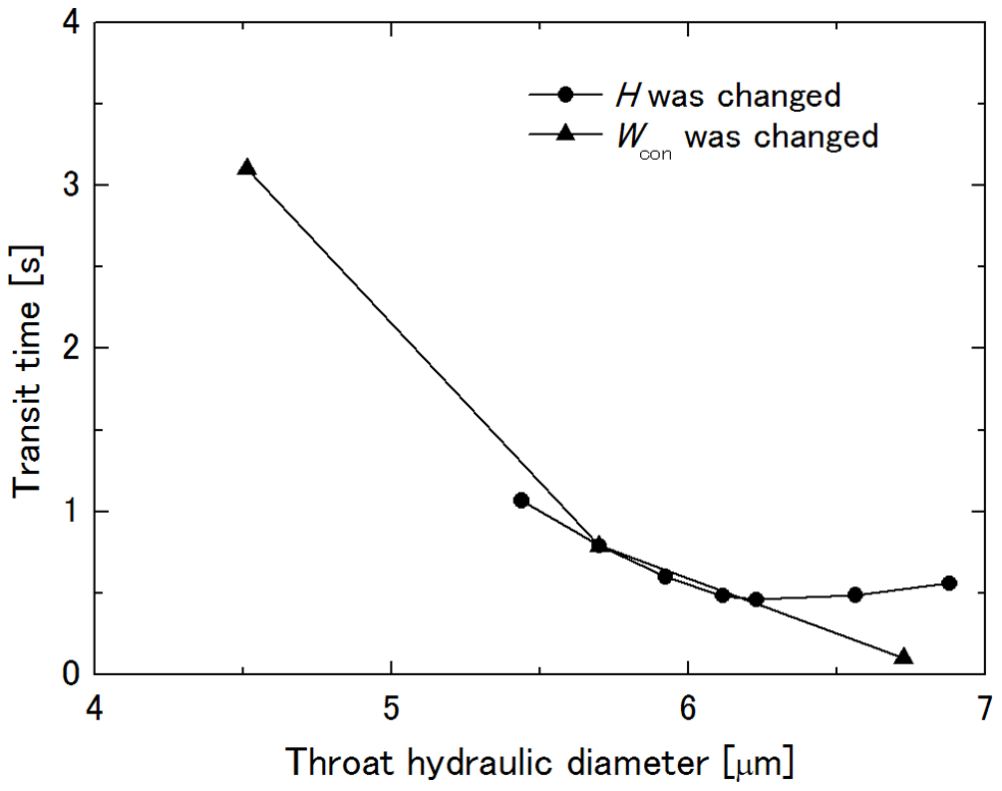
Influence of throat hydraulic diameter of constriction on transit time. *R*_con_ = 27.525 µm at Δ*P* = 40 Pa.

It is interesting that transit time through the rectangular channel was almost the same as that through the axisymmetric capillary with the same throat hydraulic diameter and that the throat aspect ratio *H*/*W*_con_ of the rectangular channel had little influence on the transit time. To examine the influence of the magnitude of cell squeezing on its transit time, Figure 10 shows the dependence of the transit time on the throat hydraulic diameter of the constriction of the rectangular channel model. Data shown here are those in Figures 5 and 6, and large throat hydraulic diameter corresponds to large *H* or *W*_con_ in those figures. It is clear that the transit time is dominated by the throat hydraulic diameter so long as the deformed cell is in contact with the ceiling and the floor of the channel during its passage through a constriction, and thus, it can be said that the magnitude of cell squeezing which correlates with the transit time can be characterized by the throat hydraulic diameter relative to the cell diameter. The same hydraulic diameter fundamentally gives the same pressure drop of a fluid across a capillary independent of the cross-sectional shape. Together with the present result that the throat aspect ratio little influences the transit time, it can be said that Dhadwal’s model [40], in which the vessel lumen of the sheet-flow model through which blood flow is characterized by its hydraulic diameter, is a good approximation to connect the sheet-flow model and the tube-flow model, and that the axisymmetric capillary model based on the tube-flow model can be replaced by the rectangular channel model based on the sheet-flow model, which has the same throat hydraulic diameter in terms of the transit time by choosing an appropriate curvature radius of the constriction. This implies applicability of rectangular microchannels to *in vitro* model experiments of neutrophil retention and passage in an alveolar capillary network.

### Limitations

Abdelgawad et al. [43] examined aspiration of SiHa cells (cervical cancer cell line) flowing from a big main channel into a narrow side branch connected at a right angle and found that the cells were trapped at the entrance of a circular channel of 5 µm in diameter at a vacuum pressure of −100 Pa while they were not trapped at a 5 µm×5 µm square channel at a vacuum pressure of −2,000 Pa. They suggested that this is due to the lack of a good seal between the cell and the channel entrance, which resulted in a leaking flow through channel corners. Therefore, it is doubtful that the present results are applicable to the passage of a cell through a channel with a sudden contraction because the sudden contraction corresponds to the limit of *R*_con_ → 0 in Figure 8 and discrepancy in the transit time between the rectangular and axisymmetric models expands with the decrease in *R*_con_. Thus, it should be noted that the present results are available for moderate constrictions. In addition, the dependence of the transit time on the throat hydraulic diameter and independence from the throat aspect ratio of the constriction are applicable within the range of a deformed cell in a constriction which is in contact with the ceiling and the floor as well as the side walls of the constriction. Otherwise, plasma leakage through clearances between the cell surface and the channel walls decreases the pressure drop across the cell to drive it, resulting in an increase in the transit time.

### Conclusions

Three-dimensional numerical simulation of the passage of a neutrophil through a rectangular channel which had a moderate constriction was performed and dependence of transit time of the cell through the constriction on the constriction geometry was investigated. Though the simulation had a limitation in the available range of the parameters, i.e., channel height, throat width and curvature radius of the constriction, for stable computations, it was found that the transit time is dominated by the throat hydraulic diameter and curvature radius of the constriction and that the throat aspect ratio little affects the transit time when the deformed cell is in contact with the ceiling and the floor as well as the side walls of the constriction. The present results suggest that, in terms of the transit time, such a rectangular channel model can be substituted for an axisymmetric capillary model having the same throat hydraulic diameter by choosing an appropriate curvature radius. Thus, microchannels fabricated by the photolithography technique are expected to be applicable to *in vitro* model experiments of neutrophil motion in an alveolar capillary network.
